# Natural Products as Adjunctive Treatment for Pancreatic Cancer: Recent Trends and Advancements

**DOI:** 10.1155/2017/8412508

**Published:** 2017-01-23

**Authors:** Qingxi Yue, Guogang Gao, Gangyong Zou, Haiqing Yu, Xi Zheng

**Affiliations:** ^1^Department of Oncology, Shanghai 9th People's Hospital, Shanghai Jiao Tong University School of Medicine, Shanghai, China; ^2^Department of Chemical Biology, Ernest Mario School of Pharmacy, Rutgers, The State University of New Jersey, Piscataway, NJ, USA; ^3^Department of Cardiothoracic Surgery, Weihai Municipal Hospital, Weihai, Shandong, China; ^4^Department of Pathology, Weihai Municipal Hospital, Weihai, Shandong, China; ^5^Department of Internal Medicine, University of Missouri Hospital and Clinics, Columbia, MO, USA; ^6^Allan H. Conney Laboratory for Anticancer Research, School of Chemical Engineering and Light Industry, Guangdong University of Technology, Guangzhou, China

## Abstract

Pancreatic cancer is a type of common malignant tumors with high occurrence in the world. Most patients presented in clinic had pancreatic cancer at advanced stages. Furthermore, chemotherapy or radiotherapy had very limited success in treating pancreatic cancer. Complementary and alternative medicines, such as natural products/herbal medicines, represent exciting adjunctive therapies. In this review, we summarize the recent advances of using natural products/herbal medicines, such as Chinese herbal medicine, in combination with conventional chemotherapeutic agents to treat pancreatic cancer in preclinical and clinical trials.

## 1. Introduction

Pancreatic cancer is a type of common malignant tumors with high occurrence in the world. Due to the high rate of invasion and malignancy and asymptomatic development, pancreatic cancer is highly lethal. Data from Global Cancer Statistics in 2012 indicated that pancreatic cancer was the seventh leading death cause from cancer in both men and women worldwide [1]. In the developed countries, pancreatic cancer was the fifth/fourth leading death cause from cancer in men and women, respectively. In the developing countries, pancreatic cancer was the eighth/tenth leading death cause from cancer in men and women, respectively [1]. The patients with pancreatic cancer had poor survival rate with less than 5% of patients surviving 5 years after diagnosis. In 2016, it was estimated that 53,070 patients will be diagnosed with pancreatic cancer and 41,780 patients with pancreatic cancer will die in the United States, most of them dying within first year of diagnosis [2]. According to the 2015 oncology annals, pancreatic cancer in China was the ninth/sixth leading death cause from cancer for men and woman, respectively. About 90,100 patients were diagnosed with pancreatic cancer and 79,400 patients in China with pancreatic cancer died in 2015 [3]. Despite recent improvements of diagnostic techniques, most pancreatic cancer patients were diagnosed at advanced states. Most patients with pancreatic cancer, who were diagnosed annually, died within a year of diagnosis. So far, there are no adequate therapies for treating the patients with pancreatic cancer.

Surgery is a pivotal curative therapeutic approach for most patients with pancreatic cancer. However, the success rate of resection surgery remains very low because about 80%–85% patients with diagnosed with pancreatic cancer were already in an advanced stage [4]. So, only 15%–20% of patients with pancreatic cancer are eligible for surgical resection after being primarily diagnosed. Standard surgical procedures include pancreaticoduodenectomy (pylorus-preserving or stomach-preserving) for tumors of pancreatic head and distal pancreatectomy with splenectomy for tumors arising in the tail or body of the pancreas [4]. Radical resection, alone or in combination with other therapy, is the only way to eradicate pancreatic cancer. Only 10%–20% of patients with pancreatic cancer, who have radical resection, can survive 5 years. So, it is necessary to underscore the need for better preoperative staging and more effective systemic therapy [5]. Therefore, chemotherapy and radiotherapy are considered as the standard treatment approaches for the patients with unresectable pancreatic cancer, especially for locally advanced or metastatic inoperable patients with pancreatic cancer.

In the past three decades, the standard therapeutic drugs for pancreatic cancer were fluoropyrimidine drug 5-fluorouracil (5-FU) and the antimetabolite drug gemcitabine [6]. Since 5-fluorouracil (5-FU) was generated 50 years ago, only the incremental changes in clinical outcomes of pancreatic cancers were made. 5-Fluorouracil (5-FU) was the first agent to be widely used to treat advanced pancreatic cancer, but the success rates of 5-FU were <20% and it was not known whether 5-FU could provide significant palliative benefits. Gemcitabine, which is now widely accepted as the golden-standard drug prescribed for the patients suffering from locally advanced (stage II or stage III) or metastatic (stage IV) pancreatic cancer, is an analog of the pyrimidine nucleotide deoxycytidine [7]. Although gemcitabine offered only an extension of ~1.5 months in median survival, gemcitabine replaced 5-fluorouracil (5-FU) as the standard first-line chemotherapeutic agent since it was approved by the Food and Drug Administration (FDA) in 1996 [7]. Current treatment modalities for the advanced pancreatic cancer include gemcitabine, as a single agent or in combination with multiple chemotherapeutic agents. Unfortunately, most patients with locally advanced and metastatic pancreatic cancers could not benefit from the monotherapy of gemcitabine. Though many clinical trials had been conducted to ascertain the optimum therapy utilizing gemcitabine in combination with multiple chemotherapeutic agents such as 5-FU, capecitabine, pemetrexed, topoisomerase inhibitors, epidermal growth factor receptor (EGFR) tyrosine kinase inhibitors, erlotinib, bevacizumab, irinotecan, exatecan, platinum compounds (cisplatin and oxaliplatin), and taxanes (paclitaxel and docetaxel), almost none of them was proven to be more effective, in comparison with gemcitabine as a single agent for treating pancreatic cancer [8]. In 2005, a four-drug regimen (gemcitabine, 5-fluorouracil, epirubicin, and cisplatin) was shown to improve the progression free survival and the overall survival of the patients with pancreatic cancer, compared with single-agent gemcitabine [8]. In 2011, a new treatment regimen FOLFIRINOX (a combination of 5-FU, leucovorin/folinic acid, oxaliplatin, and irinotecan) was demonstrated as superior survival outcomes of the patients with pancreatic cancer, compared with single-agent gemcitabine, which led to the adoption of FOLFIRINOX as the preferred option for the patients [8]. A trial comparing gemcitabine/nab-paclitaxel (nanoparticle albumin-bound paclitaxel) with gemcitabine alone showed a statistically significant survival benefit for the new doublet to introduce another option for the treatment of advanced pancreatic cancer [8].

Radiotherapy is often used to combine with systemic chemotherapy in oncology centers of USA. Radiotherapy exhibits a substantial advantage with respect to local control and improving the resectability rate after downstaging. Nonetheless, the approach has not significantly improved survival rates of the patients with unresectable pancreatic cancer. Moreover, specific radiotherapy modalities, such as stereotactic radiotherapy, TOMO, and intensity-modulated radiotherapy, have been applied to treat pancreatic cancer and survival outcomes of the patients with pancreatic cancer are partially improved [9]. However, compared with tumors in other sites, the overall survival of the patients with pancreatic cancer is unsatisfactory and the toxicity of radiotherapy is obvious. The poor prognosis of the patients with pancreatic cancer has been attributed to its late diagnosis, limitation of surgical resection, early metastases, aggressive local invasion, and robust resistance to chemotherapy and radiotherapy [9]. Hence, additional therapeutic approaches for treating pancreatic cancer are critical.

Complementary and alternative medicines, such as gene therapy, immunotherapy, targeted therapy, neoadjuvant therapy, and natural products/herbal medicines, may benefit the patients with pancreatic cancer as a supplementary therapy [10]. Of all complementary and alternative medicines, natural products/herbal medicines, such as Chinese herbal medicine, have become popular in the patients with advanced cancer, due to its efficacy and low toxicity [10]. Recent research works indicated that natural products/herbal medicines, such as Chinese herbal medicine, could provide additional strategies for monotherapy or combination treatments of various cancer types, including pancreatic cancer. Indeed, more than 60% of the current anticancer chemotherapeutic drugs used in clinic were initially developed from natural products/herbal medicines [11]. Natural products/herbal medicines, combined with conventional chemotherapy and radiotherapy, may enhance anticancer therapeutic efficacy and reduce the side effects [12]. In this context, the use of natural products/herbal medicines, as a supplementary approach, to treat pancreatic cancer holds a great promise.

Based on the published papers (up to July 15, 2016) searched in PubMed using the key words “natural product AND combination AND pancreatic cancer,” we summarized the beneficial effects of natural products/herbal medicines, such as Chinese herbal medicine, in combination with conventional chemotherapy for the patients with unresectable pancreatic cancer in preclinical and clinical trials in this review. The detailed information of the current review includes (1) combination of natural products with gemcitabine, (2) combination of natural products with other chemotherapeutic agents, (3) combination among natural products, and (4) combination of natural products in early phases of clinical trials.

## 2. Combination of Natural Products with Gemcitabine

Irofulven (MGI 114, HMAF, 6-hydroxymethylacylfulvene), a novel cytotoxic agent synthesized from the sesquiterpene mushroom metabolite of the natural product illudin S, has a unique mechanism of action involving the formation of macromolecule adduct, cell cycle arrest of S-phase, and induction of apoptosis [49]. Phase I clinical combination trial study of irofulven and gemcitabine in the patients with advanced solid tumor was underway [50]. Van Laar et al. showed similar marked activity of irofulven against pancreatic xenografts which was observed at lower total doses of an intermittent dosing regimen, compared to consecutive daily dosing [51]. Further, enhanced antitumor activity was observed when irofulven and gemcitabine were combined against the MiaPaCa pancreatic xenograft model, which indicated at least an additive interaction compared to single-agent activity. Psorospermin, a natural product isolated from the stembark and roots of the African plant* Psorospermum febrifugum*, had the activity against drug-resistant leukemia lines and AIDS-related lymphoma [52, 53]. Fellows et al. showed that psorospermin had the same effect as gemcitabine in inhibiting the growth of tumor in vivo in the MiaPaCa pancreatic xenograft model. Moreover, psorospermin combined with gemcitabine was found to have an at least additive effect in slowing the growth of MiaPaCa pancreatic cancer cells [54]. 3,3-Diindolylmethane (DIM) is a natural compound which can be easily obtained by consuming the cruciferous vegetables. Banerjee et al. present in vitro and in vivo preclinical evidence supporting chemosensitization of pancreatic cancer cells by DIM [13]. DIM potentiates chemosensitization and killing of pancreatic cancer cells by downregulation of constitutive as well as drug-induced activation of NF-kappaB and its downstream genes (XIAP, Bcl-xL, survivin, and cIAP). Compared with monotherapy, DIM pretreatment of pancreatic cancer cells resulted in a significantly increased apoptosis with suboptimal concentrations of chemotherapeutic agents such as gemcitabine, cisplatin, and oxaliplatin. Thymoquinone is a bioactive compound extracted from the oil of folklore medicine black seed (*Nigella sativa*). Banerjee et al. reported the chemosensitizing effect of thymoquinone to conventional chemotherapeutic agents (gemcitabine and oxaliplatin) both in vitro and in vivo using an orthotopic model of pancreatic cancer [14]. By downregulation of nuclear factor-kappaB (NF-kappaB/NF-*κ*B), Bcl-2 family, and NF-kappaB-dependent antiapoptotic genes (survivin, X-linked inhibitors of apoptosis, and cyclooxygenase-2), thymoquinone could potentiate the killing of pancreatic cancer cells induced by chemotherapeutic agents (gemcitabine and oxaliplatin). Cucurbitacin B, a member of the cucurbitacins, is derived from Cucurbitaceae family “*Trichosanthes kirilowii Maximowicz,*” a plant that has long been used in oriental medicine for its abortifacient, antidiabetic, and anti-inflammatory effects. Thoennissen et al. for the first time showed that cucurbitacin B has profound antiproliferative effects against human pancreatic cancer cells in vitro and in vivo and cucurbitacin B may potentate the antiproliferative activity of nucleoside analogue gemcitabine, associated with inhibition of activated JAK2/STAT3 and decrease of expression of Bcl-XL with subsequent upregulation of caspase-3 and caspase-9 [15]. Isothiocyanate sulforaphane (SF) is a natural compound present in broccoli and other cruciferous vegetables with high concentration. Kallifatidis et al. showed that SF increased the effectiveness of cytotoxic drugs (gemcitabine, cisplatin, 5-fluorouracil, and doxorubicin) against pancreatic cancer stem cells (CSCs) without inducing additional toxicity in mice [16]. Combination of SF with cytotoxic drugs intensified inhibition of spheroid formation, clonogenicity, and aldehyde dehydrogenase 1 activity along with the expression of c-Rel and Notch-1, which indicated that the characteristics of CSCs were targeted. Dimethylamino parthenolide (DMAPT) is a sesquiterpene lactone extracted from the medicinal herb feverfew (*Tanacetum parthenium*). In association with the suppression of NF-*κ*B, Holcomb et al. indicated that DMAPT enhanced the antiproliferative effects of gemcitabine in pancreatic cancer cells in vitro and in vivo, which supported the evaluation of NF-*κ*B-targeted agents to complement gemcitabine-based therapies [17]. Dimethylaminoparthenolide (DMAPT) is a novel orally bioavailable analog of parthenolide, a sesquiterpene lactone extracted from the medicinal herb feverfew (*Tanacetum parthenium*). Yip-Schneider et al. showed that the combination of DMAPT and gemcitabine significantly decreased the multiplicity and incidence of pancreatic adenocarcinomas by reducing the levels of eotaxin, tumor necrosis factor-alpha (TNF-*α*), macrophage inflammatory protein-1 beta (MIP-1*β*), inflammatory cytokines IL-12p40, and monocyte chemotactic protein-1 (MCP-1), all of which are NF-*κ*B target genes [18]. Yip-Schneider et al. also indicated DMAPT and sulindac (nonselective COX inhibitor) in combination with gemcitabine may prevent or delay the progression of premalignant pancreatic lesions in the LSL-Kras (G12D), Pdx-1-Cre mouse model of pancreatic cancer [55]. The phorbol ester, 12-O-tetradecanoylphorbol-13-acetate (TPA), is a major active ingredient extracted from the seed oil of* Croton tiglium* L., a leafy shrub of the Euphorbiaceae family which is native to Southeastern Asia.* Our laboratory* indicated that the combination of TPA with gemcitabine synergistically inhibited growth and induced apoptosis in human pancreatic cancer PANC-1 cells cultured in vitro or PANC-1 cells grown in NCr immunodeficient nude mice [19]. 0.16 nM TPA in combination with 0.5 *μ*M gemcitabine induced a remarkable increase in the expression of phosphorylated c-Jun NH2-terminal kinase (JNK) in PANC-1 cells. Guggulsterone (4,17[20]-pregnadiene-3,16-dione) is a dietary plant sterone obtained from the gum resin of the Indian Ayurvedic medicinal plant,* Commiphora mukul*, which has been used as traditional medicine since 600 BC. It has been known to show hypolipidemic activity, cardiovascular protecting activity, anti-inflammatory activity, and the activity of antagonist to bile acid receptor (farnesoid X receptor) [56]. Ahn et al. indicated that guggulsterone combined with gemcitabine augmented growth inhibition of pancreatic cancer cells in vitro and in vivo through downregulation of nuclear factor-*κ*B activity, suppression of BcL-2 and Akt expression, and activation of Bax and c-Jun NH(2)-terminal kinase [20]. Akasaka et al. indicated that monogalactosyl diacylglycerol (MGDG), a glycoglycerolipid isolated from spinach, combined with gemcitabine revealed synergistic effects of inhibitive proliferation on human pancreatic cancer cell lines BxPC-3, MiaPaCa2, and PANC-1 through the inhibition of DNA replicative pols alpha and gamma activities, compared with MGDG or GEM alone [21]. Glaucarubinone, a quassinoid natural product, was first extracted from the seeds of* Simarouba glauca* and numerous other species in the family Simaroubaceae. Yeo et al. indicated glaucarubinone and gemcitabine synergistically reduced the growth of pancreatic cancer cells in vitro and in vivo via downregulation of P21-activated serine/threonine kinases PAK1 and PAK4 [22]. Thymoquinone is a bioactive constituent isolated from the volatile oil of the black seed (*Nigella sativa*). Mu et al. showed thymoquinone pretreatment following gemcitabine treatment synergistically caused an increase of apoptosis and tumor growth inhibition in pancreatic cancer cells both in vitro and in vivo, through abrogation of Notch1, PI3K/Akt/mTOR regulated signaling pathways [23]. Namba et al. indicated zidovudine, developed from spongothymidine extracted from* Cryptotethya crypta*, resensitized gemcitabine-resistant pancreatic cancer cells to gemcitabine by inhibiting the Akt-GSK3*β*-Snail pathway [24]. Piperlongumine is a natural alkaloid/amide component extracted from the fruit of the pepper* Piper longum*. Wang et al. indicated that Piperlongumine enhanced the antitumor properties of gemcitabine in human pancreatic cancer cells in vitro and in vivo by modulating the NF-kappaB pathway [25].

Escin is a natural mixture of pentacyclic triterpene saponins extracted from the horse chestnut (*Aesculus hippocastanum*/*Aesculus wilsonii *Rehd.) seeds that has been used as an antipyretic and an analgesic agent in China. It possesses anticancer activity by induction of growth inhibition and apoptosis in many human cancer cells. Wang et al. showed escin could potentiate the efficacy of gemcitabine against human pancreatic cancer in vitro and in vivo via inactivation of NF-*κ*B and consequent inhibition of c-Myc, Bcl-2, Bcl-xL, survivin, COX-2, cyclin D1, and the activation of caspase-3 [26]. Rimmon et al. indicated that the combination of escin with gemcitabine showed only additive effect in pancreatic cancer PANC-1 cells, while escin combined with cisplatin led to a significant synergistic cytotoxic effect in PANC-1 cells by downregulating NF-*κ*B signaling pathway [27]. Gum mastic, a natural resin isolated from the leaves and stem of* Pistacia lentiscus* trees, has been extensively used as both an herbal remedy and a dietary supplement for centuries in Mediterranean and Middle Eastern countries. Huang et al. indicated gum mastic significantly potentiated antiproliferative and apoptotic effects of gemcitabine in both pancreatic cancer BxPC-3 and COLO 357 cells by increasing the expression of IkappaBalpha and Bax protein, blocking NF-kappaB activation and downregulating the expression of Bcl-2 protein [28]. Zyflamend is a polyherbal formulation comprised of ten standardized and concentrated herbal extracts (ginger, holy basil, rosemary, huzhang, oregano, turmeric, Chinese goldthread, baikal skullcap, barberry, and green tea). Kunnumakkara et al. showed Zyflamend in combination with gemcitabine synergically inhibited the growth of human pancreatic cells in vitro and in vivo by inhibiting NF-*κ*B signaling pathways [29]. MK615, called* Ume* in Japanese, is a sticky extract from Japanese apricot, which has been used to treat intestinal disorders for many years as an antipyretic and an anti-inflammatory agent [57]. Hattori et al. indicated MK615, in both the presence and absence of gemcitabine, significantly inhibited the growth of human pancreatic cancer cells in vitro and in vivo without apparent adverse effects through a reactive oxygen species-dependent mechanism [30]. Herbal preparation of Pao Pereira, obtained from a rainforest tree in the family of Apocynaceae, has long been used by practitioners and oncologic patients in complementary and alternative medicine. Yu et al. showed the combination of the extract of Pao Pereira and gemcitabine had a synergistic effect in inhibiting growth and inducing apoptosis of pancreatic cancer cells [31]. Qingyihuaji formula (QYHJ), consisting of traditional Chinese herbs, has been used for integrative treatment of human pancreatic cancer in China for many years. Xu et al. indicated that QYHJ could enhance the antitumor activity of gemcitabine pancreatic cancer cells by downregulating the expression of Jagged-1 and Notch-4 in Notch signaling pathway [32]. Pan et al. indicated that PBI-05204, a modified supercritical CO_2_ extract of* Nerium oleander*, markedly enhanced the antitumor efficacy of gemcitabine in a human pancreatic cancer PANC-1 orthotopic model and human pancreatic cancer cell lines, through downregulation of PI3k/Akt and mTOR pathways [33].

Devil's club* Oplopanax horridus* (DC), an important medicinal herb of the Pacific Northwest, is a deciduous shrub related in taxonomy to the well-known medicinal plants such as Asian ginseng (*Panax ginseng C. A. Meyer*), American ginseng (*Panax quinquefolius L.*), and eleuthero (*Eleutherococcus senticosus* Maxim. or* Acanthopanax senticosus*, formerly known as* Siberian ginseng*). The inner root and stem bark extract of DC show antiproliferation activity on multiple cancer cells in vitro. Tai et al. indicated that there was a significant antiproliferation activity of DC extract alone or in combination with chemotherapeutic agents (gemcitabine, cisplatin, and paclitaxel) on human pancreatic cancer PANC-1 3D spheroids and 2D monolayer cells [34]. Cheung et al. also indicated that DC 70% ethanol extract alone or in combination with chemotherapeutic agents (gemcitabine, cisplatin, and paclitaxel) displayed the high antiproliferation potency on pancreatic endocrine HP62 cells and pancreatic ductal carcinoma BxPC-3 and PANC-1 cells [35]. Summary of pharmacological studies of combination of natural products with gemcitabine was shown in Table 1.

## 3. Combination of Natural Products with Other Chemotherapeutic Agents

*Our laboratory* indicated that the combination of 12-O-tetradecanoylphorbol-13-acetate (TPA) with All-trans Retinoic Acid (ATRA) synergistically inhibited growth and increased apoptosis in human pancreas cancer cells cultured in vitro or pancreas tumor xenografts in immunodeficient mice [36]. The combination of TPA and ATRA induced a remarkable decrease ratio of the percentage of mitotic cells to the percentage of caspase-3-positive cells in the tumors compared with tumors from the vehicle-treated control animals.* Our laboratory* showed that the combination of TPA and diethyldithiocarbamate (DDTC) synergistically inhibited growth and increased apoptosis in human pancreas cancer cells cultured in vitro and pancreas tumor xenografts in nude mice [37]. The combination of TPA and DDTC induced a remarkable inhibition on the activation of nuclear factor-*κ*B (NF-*κ*B) and decreased the expression of Bcl-2. Parthenolide is a sesquiterpene lactone extracted from the medicinal herb feverfew (*Tanacetum parthenium*). Yip-Schneider et al. showed that the combination of nonsteroidal anti-inflammatory drug sulindac with parthenolide inhibited cell growth additively in pancreatic cancer PANC-1 cells and synergistically in pancreatic cancer BxPC-3 and MiaPaCa-2 cells, by increasing the expression level of IkappaBalpha protein and decreasing the binding and transcriptional activities of NF-kappaB DNA [38]. Yip-Schneider et al. also indicated that the combination of the cyclooxygenase 2 inhibitor celecoxib with dimethylaminoparthenolide exhibited significant inhibitory effect of tumor invasion into adjacent organs and metastasis in a carcinogen-induced pancreatic cancer model of Syrian golden hamsters, by decreasing the activity of nuclear factor-kappaB and the expression of prostaglandin E2 and prostaglandin E2 metabolite [39]. Yang et al. indicated that triptolide in combination with hydroxycamptothecin displayed the synergistic cytotoxic effect on pancreatic cancer PANC-1 cells [40]. Sulforaphane, derived from glucoraphanin, was a major glucosinolate and a dietary component in broccoli and broccoli sprouts. Li et al. indicated that the combination of sulforaphane with Hsp90 inhibitor 17-allylamino-17-demethoxygeldanamycin synergistically inhibited cell growth in vitro and in pancreatic cancer xenograft model in vivo, by decreasing the function of Hsp90 and increasing the activity of caspase-3 [41]. Yuan et al. indicated that the combination of small-molecule BRD4770 (an histone methyltransferase G9a inhibitor) with gossypol (a natural product isolated from cottonseeds) synergistically enhanced the cytotoxicity of p53-mutant pancreatic cancer PANC-1 cells but there was no effect in immortalized nontumorigenic pancreatic cells [42]. The combination of gossypol and BRD4770 induced autophagy-related cell death in pancreatic cancer cells by increasing the levels of LC3-II and the number of autophagosome.

SPES contains 15 herbs:* Cervus nippon*,* Pyrola rotundifolia*,* Panax ginseng*,* Ganoderma japonicum*,* Agrimonia pilosa*,* Cistanche deserticola*,* Corydalis bulbosa*, Pollen,* Glycyrrhiza glabra*,* Stephania delavayi*,* Lycoris radiata*,* Stephania sinica*,* Zanthoxylum nitidum*,* Rabdosia rubescens,* and* Patrinia heterophylla*. PC-SPES consists of powders derived from 8 herbs:* Dendranthema morifolium*,* Isatis indigotica*,* Panax ginseng*,* Ganoderma lucidum*,* Serenoa repens*,* Scutellaria baicalensis*,* Rabdosia rubescens*, and* Glycyrrhiza glabra*. Schwarz et al. evaluated the antitumor effects of Chinese herbs (SPES and PC-SPES) combined with the chemotherapeutic drugs (doxorubicin or gemcitabine) on eight human pancreatic cancer cell lines (CFPAC, BxPC, MIA, HS-766T, PANC-1, HTB-147, CaPan-2, and ASPC) in vitro [43]. Mediated via induction of apoptosis, both SPES and PC-SPES exhibited significant inhibitory effect on pancreatic cancer cells. Combination effects with either extract appeared to be additive to mildly synergistic in the case of gemcitabine while they appeared to be antagonistic in the case of doxorubicin.* Berberis vulgaris*, which belongs to Berberidaceae family, is a plant growing in Asia and Europe.* Berberis* extract has been used for a long time in folk medicine, whose therapeutic value is attributed to the fruits, leaves, bark, and root. Berberine is the most potent of these alkaloids extracted from* Berberis vulgaris*, responsible for the majority of pleiotropic effects against a number of cancer cell lines. Issat et al. showed cholesterol-reducing agents lovastatin in combination with berberine exert potentiated cytotoxic and/or cytostatic effects against murine Panc 02 pancreatic cancer cells and human MDA-MB231 breast cancer cells [58]. Cell cycle was inhibited in G1 phase after 48 h of incubation with lovastatin and berberine. The combination of lovastatin and berberine slightly, but significantly, decreased tumor growth in a Panc 02 pancreatic cancer model of mice.* Moringa oleifera *Lam. (Moringaceae) is a tree that grows widely in the tropics and subtropics of Asia and Africa. Leaves of* Moringa oleifera* consist of flavonoid pigments, such as kaempferitrin, kaempferol, isoquercitrin, and rhamnetin. Berkovich et al. indicated that* Moringa oleifera* leaf extract synergistically inhibited tumor growth and enhanced the cytotoxic effect of cisplatin in human pancreas cancer PANC-1 cells in vitro, by elevating the sub-G1 cell population of cell cycle and reducing the expression of p65, p-IkB*α*, and IkB*α* proteins [44]. Reis et al. indicated that the combination of lathyranes, the chemical compound isolated from Euphorbia piscatoria, and doxorubicin synergistically enhanced the antiproliferative activity on human pancreatic cells in vitro [59]. Summary of pharmacological studies of combination of natural products with other chemotherapeutic agents was shown in Table 2.

## 4. Combination between Natural Products

Mohammad et al. showed that the combination of (−)-gossypol (a natural polyphenolic compound extracted from cotton seeds) with genistein (a prominent soy isoflavone) more significantly inhibited the growth of BxPC-3 pancreatic cancer cells, compared with either agent alone. Genistein, which inactivated NF-kB and caused transcriptional inactivation of Bcl-XL and Bcl-2, could be combined with (−)-gossypol to inactivate the function of Bcl-XL and Bcl-2 and then enhanced the death of pancreatic cancer cells [45]. Srivastava et al. indicated that sulforaphane, an active compound in cruciferous vegetables, synergistically inhibits self-renewal capacity of pancreatic cancer stem cells with quercetin, a major polyphenol and flavonoid commonly detected in many fruits and vegetables, by inhibiting the expression of Nanog, phosphorylation of FKHR, Bcl-2, XIAP, activating caspase-3, and proteins involved in the epithelial-mesenchymal transition (beta-catenin, twist-1, ZEB1, and vimentin) [46]. Ding et al. showed the combination of that wogonin, a naturally occurring flavone, with the structurally related natural flavones apigenin and chrysin could enhance TRAIL-mediated apoptosis in pancreatic carcinoma CaPan-1 cells, by upregulation of TRAIL receptor 2 (TRAIL-R2) and transcriptional downregulation of c-FLIP (a key inhibitor of death receptor signaling) [47]. Yue et al. indicated that metformin combined with aspirin synergistically inhibited tumor growth, migration, and colony formation in human pancreas cancer PANC-1 and BxPC-3 cells cultured in vitro and pancreas tumor xenografts in nude mice, by remarkably inhibiting the phosphorylation of STAT3 and mTOR, significantly downregulating the antiapoptotic proteins Bcl-2 and Mcl-1 and significantly upregulating the proapoptotic proteins Puma and Bim [48]. Summary of pharmacological studies of combination between natural products was shown in Table 3.

## 5. Combination of Natural Products in Early Phases of Clinical Trials

Hauns et al. indicated that* Ginkgo biloba* extract GBE 761 ONC combined with 5-fluorouracil (5-FU) was shown favourable effect to treat the patients with pancreatic cancer in the clinical trial phase II study, compared to the clinical trial of 5-FU monotherapy [60]. Curcumin, which is called diferuloyl methane, is a hydrophobic polyphenol isolated from rhizome (turmeric) of the herb* Curcuma longa*. Curcumin has shown various activities, such as a mediator of chemoresistance and radio-resistance, antioxidant, anti-inflammatory, and immunomodulatory activities, enhancing of the apoptotic process, cytokines release, and antiangiogenic properties. The anticancer effect has been seen in a few clinical trials, mainly as a native chemoprevention agent in colon and pancreatic cancer, cervical neoplasia, and Barrett's metaplasia [61]. Although curcumin may potentiate the antitumor effect of gemcitabine by intervening with several intracellular signal transduction pathways in pancreatic cancer cells in vitro and in vivo, Epelbaum et al. showed that a combination of gemcitabine and oral curcumin at a dose of 8,000 mg/day to treat the patients with advanced pancreatic cancer is not feasible and 29% patients had to stop oral curcumin due to gastrointestinal toxicity [62]. The preliminary results suggest that a combination of curcumin and gemcitabine for the patients with advanced pancreatic cancer is feasible. Mistletoe extracts from the medicinal herb* Viscum album *L. are widely used to treat cancer patients in Europe. Matthes et al. indicated that mistletoe extract (Iscador®, Weleda, Arlesheim, Switzerland) could be supportive care in an adjuvant chemotherapy setting with gemcitabine or 5-fluorouracil (5-FU) in the patients undergoing curative intent resection of pancreatic cancer [63]. Löhr et al. reported the phase Ib study of 16 chemotherapy-native patients with inoperable pancreatic carcinoma treated with gemcitabine and AXP107-11, the sodium salt dihydrate form of genistein (genistein-SSDH, a novel multicomponent crystalline form of genistein) [64]. The results demonstrated that treatment of pancreatic cancer patients with AXP107-11 in combination with gemcitabine led to a favorable pharmacokinetics with high serum levels without toxicity.

Traditional Chinese herbal formulation Huang-Qin-Tang (HQT), which had been documented for almost 1800 years to treat common gastrointestinal distress, such as diarrhea, headache, abdominal spasms, nausea, fever, subcardiac distention, extreme thirst, and vomiting, included four distinct herbs: the roots of* Scutellaria baicalensis *Georgi. (scute),* Glycyrrhiza uralensis* Fisch. (licorice), and* Paeonia lactiflora *Pall. (peony) and the fruit of* Ziziphus jujuba *Mill. (Chinese date). Each herb of Huang-Qin-Tang had an unusual pharmacological profile, which was involved in antiviral and anticancer activity, liver protection, hematological and immunological modulation, appetite improvement, and analgesic activity. Botanical formulation PHY906, a pharmaceutical grade of Huang-Qin-Tang, was explored as an adjuvant for chemotherapy and targeted therapy of tumors by the teams led by Professor Yung-Chi Cheng in Yale University School of Medicine and PhytoCeutica, Inc. PHY906 was distinguished from Huang-Qin-Tang which is currently available in the market, due to the unique and defined procedures for its characterization, preparation, and quality control. An open label phase I/II study of PHY906 in combination with capecitabine in the patients with advanced pancreatic was concluded at Yale Cancer Center in 2009. Saif et al. conducted phase I study in the patients with advanced pancreatic and gastrointestinal malignancies using PHY906 combined with capecitabine to determine the maximum tolerated dose (MTD) of capecitabine in combination with PHY906 [65]. The results of phase I study indicated that the MTD of capecitabine was 1500 mg/m^2^ BID administered in a 7/7 schedule, combined with PHY906 800 mg BID on days 1–4. Saif et al. also carried out phase II study in the patients with advanced pancreatic cancer who were previously treated with gemcitabine-based regimens to explore the efficacy of capecitabine in combination with PHY906 [66]. The results of phase II study showed that capecitabine combined with PHY906 displayed a feasible and safe salvage therapy after the failure of gemcitabine for advanced pancreatic cancer.

## 6. Conclusions and Future Directions

Although there are great improvements in the treatment of many common cancers in clinic (e.g., prostate cancer and breast cancer) in recent decades, pancreatic cancer still remains the most deadly diagnosed cancer and represents a major challenge [10]. Herbal remedies have been used to treat various diseases for thousands of years in numerous countries, such as China, Egypt, Japan, and Korea, which have come to be accepted as forms of complementary and alternative medicines in western countries [67]. Therefore, natural products/herbal medicines play important roles in prevention and therapy of pancreatic cancer as a promising adjunctive approach. They have been credited with substantial advantages, including improving immune system, suppressing tumor progression, enhancing beneficial effects, and lessening adverse/side effects of chemotherapy and radiotherapy [11]. Unlike western medicine which generally uses purified chemical compounds and targets single physiological endpoints, natural products/herbal medicines usually consist of multiple components and herbs which act/interact simultaneously through various cellular signal mechanisms and molecular targets [10]. These multiple herbs serve various functions; some may improve the efficacy while others may increase the bioavailability or decrease the toxicity. Even isolated compounds from natural products/herbal medicines may display multiple effects, such as improving the efficacy and decreasing the toxicity of chemotherapy and/or radiotherapy. So, there are various natural products/herbal medicines targeting multiple cancer-related proteins and pathways, which make them an attractive direction as supplementary therapeutics for the patients with pancreatic cancer [11].

Herbal medicine formulations, including Chinese herbal medicine, have been originated from empirical observations in humans over thousands of years. As a complementary and alternative therapy, Chinese herbal medicine has increasingly drawn an interest among international cancer research studies because it can increase the efficacy and decrease the toxicity when combined with chemotherapy and/or radiotherapy. Moreover, according to NCCN clinical practice guidelines, the integration of palliative care in cancer patients has become standard oncology practice when a patient is diagnosed with metastatic or advanced cancer, including pancreatic cancer [12]. Chinese herbal medicine has a longstanding history in China and it is deeply embedded in urban and rural populations as a measure of palliative care. The phenomena are attributed to the fact that the origin and the development of traditional Chinese medicine are intricately entwined with Chinese history, economy, politics, and culture, whose compelling efficacy has been attested. Though Chinese herbal medicine has multiple complicated components and probable adverse effects, its application has been widely embraced in clinical practice, especially throughout China. Chinese herbal medicine has also been accepted into Chinese clinical practice guidelines to treat pancreatic cancers [12]. Preclinical studies have demonstrated that Chinese herbal medicine can suppress tumor proliferation and metastasis. Recent reported studies have shown that 90% Chinese patients with cancer are treated with diverse Chinese herbal medicine alone or in combination with chemotherapy and/or radiotherapy during their treatment regimen [12]. Chinese herbal medicine can increase the effect of antitumor therapies and improve the quality of life or the performance status of pancreatic cancer patients, which will provide more evidence to promote the application of Chinese herbal medicine to benefit the patients with pancreatic cancer in the world.

## Figures and Tables

**Table 1:** Summary of pharmacological studies of combination of natural products with gemcitabine.

Combination of natural products with gemcitabine	Experimental model	Anticancer/anticarcinogenic effects	Mechanism of action (↓downregulated) (↑upregulated)	Reference
Combination of 3,3-diindolylmethane with gemcitabine	PANC-1 Panc-28, and Colo-357 pancreatic cancer cells (in vitro); PANC-1 pancreatic cancer cells orthotopic xenografts (in vivo)	Reduced the proliferation of pancreatic cancer cells	NF-*κ*B↓, phospho-p65↓, Bcl-xL↓, XIAP↓, XIAP↓, survivin↓, PARP cleavage↑	[13]

Combination of thymoquinone with gemcitabine	BxPC-3 and HPAC pancreatic cancer cells (in vitro); HPAC pancreatic cancer cells orthotopic xenografts (in vivo)	Reduced the proliferation of pancreatic cancer cells	NF-*κ*B↓, Bcl-2 family↓, XIAP↓, survivin↓, COX-2↓	[14]

Combination of cucurbitacin B with gemcitabine	MiaPaCa-2, PL45, PANC-1, SU86.86, AsPC-1, Panc-03.27, and Panc-10.05 pancreatic cancer cells (in vitro); PANC-1 pancreatic cancer cells xenografts (in vivo)	Synergistically potentiated the antiproliferative effects of pancreatic cancer cells	JAK2↓, STAT3↓, STAT5↓, cyclin A↓, cyclin B1↓, Bcl-XL↓, p21(WAF1)↑, p53↑, caspase-3↑, caspase-9↑	[15]

Combination of sulforaphane with gemcitabine	MIA-PaCa2 pancreatic cancer cells (in vitro); MIA-PaCa2 pancreatic cancer cells xenografts (in vivo)	Enhanced additive cytotoxic effect to pancreatic cancer cells	ALDH1↓	[16]

Combination of dimethylaminoparthenolide with gemcitabine	BxPC-3, PANC-1, and MiaPaCa-2 pancreatic cancer cells (in vitro); MiaPaCa-2 pancreatic cancer cells heterotopic xenograft (in vivo)	Synergistically inhibited the proliferation of pancreatic cancer cells	NF-*κ*B↓, I*κ*B*α*↑	[17]

Combination of dimethylaminoparthenolide with gemcitabine	MiaPaCa pancreatic cancer cells (in vitro); MiaPaCa pancreatic cancer cells xenografts (in vivo)	Synergistically inhibited the proliferation of pancreatic cancer cells	IL-12p40↓, MCP-1↓, MIP-1*β*↓, eotaxin↓, TNF-*α*↓	[18]

Combination of 12-O-tetradecanoylphorbol-13-acetate with gemcitabine	PANC-1 pancreatic cancer cells (in vitro); PANC-1 tumors grown in immunodeficient mice (in vivo)	Synergistically inhibited the growth and induced apoptosis in PANC-1 cells	JNK↑	[19]

Combination of guggulsterone with gemcitabine	MiaPaCa-2 and Panc-1 pancreatic cancer cells (in vitro); MiaPaCa-2 pancreatic cancer cells xenografts (in vivo)	Synergistically enhanced antitumor efficacy of pancreatic cancer cells through apoptosis induction	NF-*κ*B↓, Akt↓, BcL-2↓, c-Jun↑, Bax↑	[20]

Combination of monogalactosyl diacylglycerol with gemcitabine	BxPC-3, MiaPaCa2 and PANC-1 pancreatic cancer cells (in vitro)	Synergistically enhanced the growth suppression of pancreatic cancer cells	Mammalian pol *α*↓, *γ*↓, *δ*↓ and *ε*↓	[21]

Combination of glaucarubinone with gemcitabine	PANC-1, MiaPaCa-2, and PAN02 pancreatic cancer cells (in vitro); PANC-1 and MiaPaCa-2 pancreatic cancer cells xenograft (in vivo)	Inhibited the growth of pancreatic cancer cells	PAK1↓, PAK4↓	[22]

Combination of thymoquinone with gemcitabine	PANC-1, BxPC-3, and AsPC-1 pancreatic cancer cells (in vitro); PANC-1 pancreatic cancer cells orthotopic xenograft (in vivo)	Synergistically caused an increase in pancreatic cancer cells apoptosis and tumor growth inhibition both in vitro and in vivo	Notch1↓, NICD↓, PTEN↑, p-p65↓, Bcl-2↓, Bcl-xL↓, XIAP↓, caspase-3↑, caspase-9↑, Bax↑, cytochrome c↑	[23]

Combination of Zidovudine with gemcitabine	PK1 and KLM1 pancreatic cancer cells (in vitro); PK1 and KLM1 pancreatic cancer cells xenografts (in vivo)	Resensitized gemcitabine-resistant pancreatic cancer cells to gemcitabine	hENT1↓, EMT-like phenotype↑, the Akt-GSK3*β*-Snail1 pathway↑	[24]

Combination of piperlongumine with gemcitabine	PANC-1, BxPC-3, and AsPC-1 pancreatic cancer cells (in vitro); BxPC-3 pancreatic cancer cells xenografts (in vivo)	Synergistically inhibited the proliferation of pancreatic cancer cells	NF-*κ*B↓, c-Myc↓, cyclin D1↓, Bcl-2↓, Bcl-xL↓, survivin↓, XIAP↓, VEGF↓, MMP-9↓, PCNA↓, Ki-67↓, CD31↓	[25]

Combination of escin with gemcitabine	BxPC-3 and PANC-1 pancreatic cancer cells (in vitro); BxPC-3 pancreatic cancer cells xenografts subcutaneously established in BALB/c nude mice (in vivo)	Dramatically enhanced the suppressive effect of pancreatic cancer cells	NF-*κ*B↓, c-Myc↓, COX-2↓, Cyclin D1↓, Survivin↓, Bcl-2↓, Bcl-xL↓, caspase-3↑	[26]

Combination of escin with gemcitabine	PANC-1 pancreatic cancer cells (in vitro)	Enhanced additive cytotoxic effect to pancreatic cancer cells	NF-*κ*B↓, cyclin D↓	[27]

Combination of gum mastic with gemcitabine	BxPC-3 and COLO 357 pancreatic cancer cells (in vitro)	Enhanced antiproliferative and apoptotic effects of pancreatic cancer cells	NF-*κ*B↓, I*κ*B*α*↑, Bax↑, Bcl-2↓	[28]

Combination of Zyflamend with gemcitabine	AsPC-1, BxPC-3, MiaPaCa-2, and PANC-1 pancreatic cancer cells (in vitro); MiaPaCa-2 pancreatic cancer cells orthotopic xenografts (in vivo)	Inhibited the growth of human pancreatic tumors and sensitized pancreatic cancer to gemcitabine	NF-*κ*B↓, cyclin D1↓, c-myc↓, COX-2↓, Bcl-2↓, IAP↓, survivin↓, VEGF↓, ICAM-1↓, CXCR4↓, Ki-67↓, COX-2↓, MMP-9↓	[29]

Combination of Japanese apricot extract (MK615) with gemcitabine	MiaPaCa-2 pancreatic cancer cells (in vitro); MiaPaCa-2 pancreatic cancer cells xenografts (in vivo)	Significantly inhibited the growth of human pancreatic cancer cells	ROS↓	[30]

Combination of the extract of Pao Pereira with gemcitabine	PANC-1, AsPC-1, HPAF-II, BxPC-3, and MiaPaCa-2 pancreatic cancer cells (in vitro); PACN-1 pancreatic cancer cells xenografts (in vivo)	Enhanced the inhibitory effect of pancreatic cancer cells	Caspase-8↑, caspase-3↑, PARP↑	[31]

Combination of Qingyihuaji formula with gemcitabine	SW1990 pancreatic cancer cells (in vitro); SW1990 pancreatic cancer cells xenografts (in vivo)	Enhanced the antitumor activity of gemcitabine to pancreatic cancer cells	Notch-4↓, Jagged-1↓, CD133↓	[32]

Combination of PBI-05204 (a supercritical CO_2_ extract of *Nerium oleander *containing oleandrin) with gemcitabine	PANC-1 pancreatic cancer cells (in vitro); PANC-1 pancreatic cancer orthotopic model (in vivo)	Markedly enhanced the antitumor efficacy of gemcitabine to pancreatic cancer cells	Ki-67↓, pAkt↓, pS6↓, p4EPB1↓	[33]

Combination of Devil's club *Oplopanax horridus* with gemcitabine	PANC-1 pancreatic cancer cells (in vitro)	Significantly enhanced the antiproliferation activity of gemcitabine to pancreatic cancer cells	Bcl-2↓, BAX↑, caspase-3↑	[34]

Combination of Devil's club *Oplopanax horridus* with gemcitabine	PANC-1 and BxPC-3 pancreatic cancer cells (in vitro)	Significantly enhanced the antiproliferation activity of gemcitabine to pancreatic cancer cells	Cytochrome C↑, claspin↑, cIAP-2↑	[35]

**Table 2:** Summary of pharmacological studies of combination of natural products with other chemotherapeutic agents.

Combination of natural products with other chemotherapeutic agents	Experimental model	Anticancer/anticarcinogenic effects	Mechanism of action (↓downregulated) (↑upregulated)	Reference
Combination of 12-O-tetradecanoylphorbol-13-acetate with all-trans retinoic acid	PANC-1, MiaPaCa-2, and BxPC-3 pancreatic cancer cells (in vitro); PANC-1 tumor xenografts in immunodeficient mice (in vivo)	Enhanced the inhibitory effect to pancreatic cancer cells	p21↑, caspase-3↑	[36]

Combination of 12-O-tetradecanoylphorbol-13-acetate with diethyldithiocarbamate	PANC-1 pancreatic cancer cells (in vitro); PANC-1 tumor xenografts in nude mice (in vivo)	Significantly inhibited the growth of pancreatic cancer cells	NF-*κ*B↓, Bcl-2↓	[37]

Combination of parthenolide with sulindac	BxPC-3, PANC-1, and MiaPaCa-2 pancreatic cancer cells (in vitro)	Synergistically inhibited the growth of MiaPaCa-2 and BxPC-3 cells and additively inhibited the growth of PANC-1 cells	NF-*κ*B↓, I*κ*B*α*↑	[38]

Combination of celecoxib with dimethylaminoparthenolide	PC-1 pancreatic cancer cells xenografts (in vivo)	Significantly inhibited the growth of pancreatic cancer cells	NF-*κ*B↓, prostaglandin E2↓, prostaglandin E2 metabolite↓	[39]

Combination of triptolide with hydroxycamptothecin	MiaPaCa pancreatic cancer cells (in vitro); MiaPaCa pancreatic cancer cells xenografts (in vivo)	Enhanced synergistic cytotoxic effect to pancreatic cancer cells	NF-*κ*B↓, caspase-9↑, caspase-3↑	[40]

Combination of sulforaphane with 17-allylamino 17-demethoxygeldanamycin	MiaPaCa-2 pancreatic cancer cells (in vitro)	Enhanced the inhibitory effect to pancreatic cancer cells	Caspase-3↑, Hsp90↓	[41]

Combination of gossypol with BRD4770 (an HMT G9a inhibitor)	PANC-1 pancreatic cancer cells (in vitro)	Enhanced the cytotoxicity to p53-mutant PANC-1 cells in a synergistic manner	LC3-II↑, autophagosome↑	[42]

Combination of Chinese herbs SPES with PC-SPES	MIA, PANC-1, BxPC, ASPC, HS-766T, CaPan-2, CFPAC, and HTB-147 pancreatic cancer cells (in vitro)	Exhibited significant toxicity to pancreatic cancer cells		[43]

Combination of Moringa Oleifera aqueous leaf extract with cisplatin	PANC-1, p34 and COLO 357 pancreatic cancer cells (in vitro)	Synergistically enhanced the cytotoxic effect to pancreatic cancer cells	p65↓, p-IkB*α*↓, IkB*α*↓	[44]

**Table 3:** Summary of pharmacological studies of combination between natural products.

Combination between natural products	Experimental model	Anticancer/anticarcinogenic effects	Mechanism of action	Reference
Combination of (−)-gossypol with genistein	BxPC-3 pancreatic cancer cells (in vitro)	Significantly inhibited the growth of pancreatic cancer cells	Bcl-XL/Bim heterodimerization↓, NF-*κ*B↓, Bcl-2↓, Bcl-XL↓	[45]

Combination of sulforaphane with quercetin	Pancreatic cancer stem cells (in vitro)	Significantly eliminated the growth of pancreatic cancer stem cells	Bcl-2↓, XIAP↓, p-FKHR↓, caspase-3↑, beta-catenin↓, vimentin↓, twist-1↓, ZEB1↓	[46]

Combination of wogonin with apigenin and chrysin	CaPan-1 pancreatic carcinoma cells (in vitro)	Enhanced TRAIL-mediated apoptosis of CaPan-1 human pancreatic carcinoma cells	c-FLIP↓, p53↑, Mdm2↓, TRAIL-R2↑	[47]

Combination of metformin with aspirin	PANC-1 and BxPC3 pancreatic carcinoma cells (in vitro); PANC-1 xenograft mouse model (in vivo)	Significantly inhibit the growth of pancreatic cancer cells	p-mTOR↓, p-STAT3↓, caspase-3↑, PARP cleavage↑, Mcl-1↓, Bcl-2↓, Bim↑, Puma↑	[48]
